# Percutaneous intervertebral bridging cementoplasty for adjacent multilevel osteoporotic thoracolumbar fractures with vertebral endplate-disc complex injury: technical note

**DOI:** 10.1038/s41598-020-71343-w

**Published:** 2020-09-01

**Authors:** Song Wang, Chunyan Duan, Han Yang, Jianping Kang, Qing Wang

**Affiliations:** 1 Department of Orthopaedics, The Affiliated Hospital of Southwest Medical University, No. 25 Taiping St., Luzhou, 646000 Sichuan China; 2 School of Basic Medical Sciences, Southwest Medical University, Luzhou, 646000 Sichuan China

**Keywords:** Trauma, Outcomes research

## Abstract

This paper describes a minimally invasive technique of percutaneous intervertebral bridging cementoplasty (PIBC) to augment the fractured vertebrae and immobilize the intervertebral space with endplate-disc complex injury simultaneously. Thirty-two patients with adjacent multilevel osteoporotic thoracolumbar fractures (AMOTLFs) and vertebral endplate-disc complex injury (EDCI) treated by PIBC were retrospectively reviewed. The PIBC technique was a combination of puncture, balloon expansion and bridging cementoplasty. The clinical and radiological assessments were reviewed. The operation time was 82.8 ± 32.5 min, and blood loss was 76.9 ± 31.7 mL. A cement bridge was connected between the two fractured vertebrae across the injured intervertebral space. VAS at three time points including pre-operation, post-operation 1 day and final follow-up was 6.9 ± 0.9, 2.9 ± 0.8 and 1.7 ± 0.8, respectively; ODI at three time points was (71.1 ± 7.8)%, (18.4 ± 5.7)%, and (10.3 ± 5.7)%, respectively; Cobb angle at three time points was 46.0° ± 10.4°, 25.9° ± 8.5°, and 27.5° ± 7.1°, respectively. Compared with pre-operation, VAS, ODI and Cobb angle were significantly improved at post-operation 1 day and final follow-up (P < 0.05). Clinical asymptomatic cement leakage was observed in thirteen patients. No vessel or neurological injury was observed. PIBC may be an alternative way of treatment for AMOTLFs with EDCI. The technique is a minimally invasive surgery to augment the fractured vertebrae and immobilize the injured intervertebral space simultaneously.

## Introduction

Osteoporotic vertebral fracture is an increasing common spinal disorder among the elderly patients. Thoracolumbar vertebrae are frequently involved segments and they can cause disabling pain and kyphotic deformity^1^. Although conservative pain management is recommended for some patients, minimally invasive vertebral augmentation is generally advocated for symptomatic osteoporotic vertebral fracture^2,3^.

Patients with symptomatic acute or subacute osteoporotic vertebral compression fracture (OVCF) are often considered potential candidates for treatment with vertebral augmentation. Percutaneous vertebroplasty (PVP) and percutaneous kyphoplasty (PKP) are the preferred augmentation techniques which provided rapid pain relief and sustained improvement of physical function^3,4^. Furthermore, PKP has advantages of correcting the kyphotic deformity and restoring the height of the fractured vertebrae. However, some OVCFs are characterized not only by vertebral compression fractures, but also by vertebral endplate-disc complex injury (EDCI)^5^. Current treatment strategies, such as vertebroplasty and kyphoplasty, are aimed only at stabilizing these painful vertebral fractures. EDCI cannot be treated by traditional augmentation techniques. Consequently, EDCI may account for cement leakage into the disc and persistent back pain after vertebral augmentation. In addition, EDCI may be labeled as new adjacent levels fracture or instability^6^.

OVCF with EDCI is not rare. Ortiz et al.^5^ reported that about 80% patients with OVCF showed an association with vertebral endplate and disc injury as seen on MR images of the thoracic and lumbar spine. Currently, improved pedicle screw fixation and fusion techniques are being used for the management of osteoporotic vertebral fractures with segmental instability, including expandable pedicle screws and cement augmented-pedicle screws^7,8^. These methods could enhance the fixation strength by increasing the pedicle screw interface and pullout force in osteoporotic vertebra. However, the surgical trauma and the associated complications are still a concern^9,10^. Moreover, it may be not suitable for patients with very severe osteoporosis and those with associated cardiopulmonary diseases.

In this study, 32 patients diagnosed with adjacent multilevel osteoporotic thoracolumbar fractures (AMOTLFs) and EDCI were treated by percutaneous intervertebral bridging cementoplasty (PIBC). We aim at describing this minimally invasive technique to augment the fractured vertebrae and immobilize EDCI simultaneously.

## Patients and methods

### Patients

**Figure 1:**
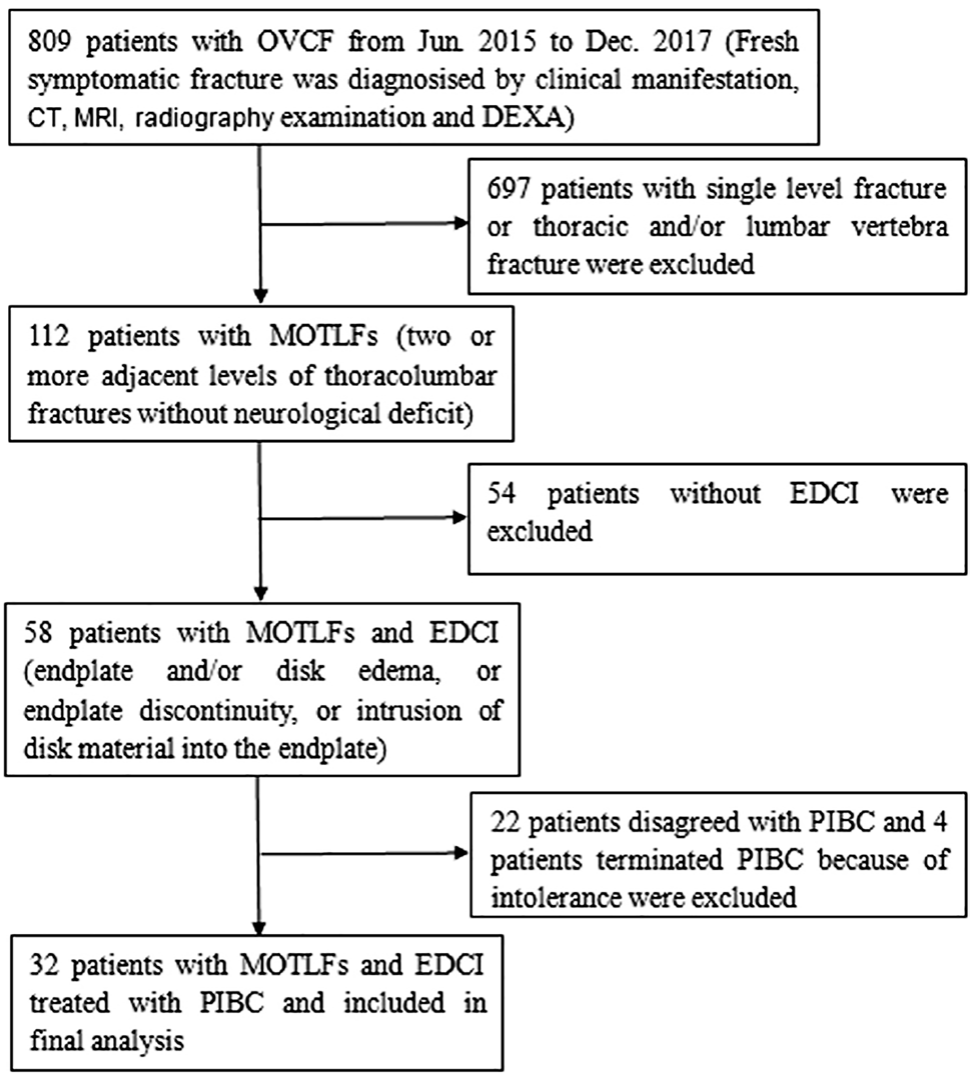
The patient selection flow chart.

We retrospectively reviewed 32 patients with AMOTLFs and EDCI who were treated with PIBC from June 2015 to December 2017. They met the following inclusion criteria: (1) relevant osteoporosis (T-Score less than − 2.5 SD) by dual energy X-ray absorptiometry (DEXA); (2) two or more adjacent levels of thoracolumbar fractures without neurological deficit; (3) EDCI between the fractured thoracolumbar vertebrae determined by the presence of endplate and/or disc edema, morphologic alteration, endplate discontinuity, or intrusion of disc material into the endplate. Patients with Spinal tumor or infectious spondylitis were excluded. The patient selection flow chart is shown in Fig. 1. Among these patients, 32 patients with AMOTLFs and EDCI were included. Four patients were finally excluded because they cannot tolerate puncture under local anesthesia. And then nerve block therapy around facet joint was performed. Patient characteristics are listed in Table 1.

**Table 1:** Patient demographic and baseline characteristics.

No.	Age (years)/sex	Cause of fracture	Course (days)	BMD	Hospital day (days)	FU (ms)
1	54/F	VA	3	2.5	2	6
2	93/F	NCT	180	4.2	15	54
3	65/F	NCT	32	2.8	4	24
4	71/M	SLI	30	3.5	3	48
5	73/F	Fall	14	3.8	5	24
6	69/F	SLI	15	4	6	36
7	56/F	NCT	18	2.5	10	36
8	59/F	Fall	7	2.8	7	9
9	64/F	SLI	3	3	3	12
10	63/F	VA	5	3	10	15
11	62/M	SLI	8	3.2	7	18
12	73/M	SLI	10	4.1	6	21
13	80/F	Fall	20	4.5	3	24
14	82/M	NCT	60	3.4	4	24
15	81/F	SLI	80	3.5	7	27
16	54/F	NCT	150	2.5	6	30
17	91/F	SLI	40	2.2	3	30
18	73/M	NCT	21	2.4	9	36
19	65/M	SLI	14	3.5	8	39
20	60/F	SLI	30	3.8	6	42
21	66/F	SLI	33	3.9	5	48
22	71/F	NCT	60	2.6	3	51
23	75/M	NCT	70	2.8	4	28
24	78/M	NCT	80	3	5	48
25	74/F	NCT	100	2.6	3	24
26	63/F	SLI	7	3.5	6	51
27	67/M	NCT	150	3	8	9
28	69/F	Fall	14	3.1	7	12
29	70/M	SLI	16	2.8	3	24
30	73/F	SLI	30	2.5	5	24
31	75/M	NCT	10	3	4	48
32	68/F	SLI	5	2.9	3	36

*BMD* bone mineral density, *FU* follow-up, *ms* months, *F* female, *M* male, *VA* vehicle accident, *SLI* slight life injury, *NCT* no complained trauma.

Plain radiographs and computed tomography (CT) with three-dimensional reconstruction in all patients were obtained at preoperatively, one day postoperatively, and at the final follow-up. Magnetic resonance imaging (MRI) was performed preoperatively in all patients and postoperatively readmitted patients. All the images were reviewed and analyzed by our team. In addition, the comorbidities, operation time, blood loss, hospital stay and complications were recorded and reviewed carefully. The clinical outcomes were assessed in terms of thoracolumbar (T10–L2) kyphotic Cobb angle (TLK), back pain visual analog scale (VAS), and Oswestry disability index (ODI) preoperatively, one day postoperatively, and at the final follow-up. The outcome assessments (TLK, VAS, and ODI) were expressed as mean ± SD and compared with paired *t* test using GraphPad Prism 6 software (GraphPad Software Inc, San Diego, CA, USA). P < 0.05 was considered to be statistically significant.

The involved patients underwent PIBC by the same surgery team, which had a combined experience of 25 years in spinal surgery, 14 years in PVP/PKP and 6 years in PIBC. The study protocol was approved by the ethics committee of the Affiliated Hospital of Southwest Medical University. The methods were carried out in accordance with the relevant guidelines and regulations of Good Clinical Practice (GCP). Informed consent was obtained from each participant before their inclusion in the study.

### Surgical technique

The patient was placed in prone position with vacated abdomen. The procedure was performed under local anesthesia and guided by C-arm fluoroscopy. The PIBC was a combination of puncture, balloon expansion and bridging cementoplasty, which included four continuous steps.

**Figure 2:**
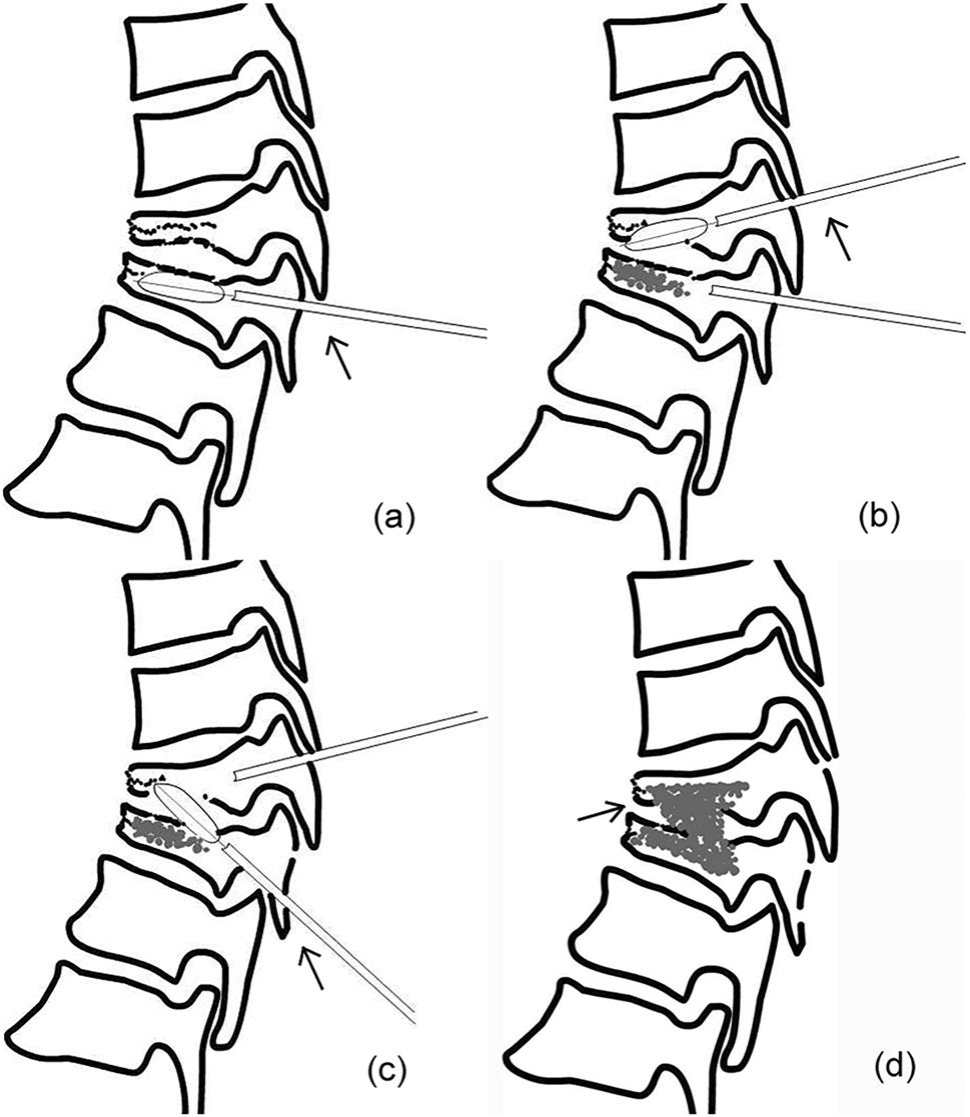
The four steps of PIBC. (**a**) Puncture and expansion for the caudal adjacent fractured vertebra (arrow); (**b**) puncture and expansion for the cranial adjacent fractured vertebra (arrow); (**c**) puncture and expansion for the intervertebral space with endplate-disc complex injury (arrow); (**d**) intervertebral bridging cementoplasty for the spinal unit (arrow).

Step 1 was the puncture and expansion for the caudal adjacent fractured vertebra (Fig. 2a). In particular, modified unilateral transpedicular puncture was used and the target puncture point of the needle was in the anterior one-third middle point of the fractured vertebral body. After the vertebral body was expanded with a balloon, the cement was injected into the anterior vertebral body.

Step 2 was the puncture and expansion for the cranial adjacent fractured vertebra (Fig. 2b). Subsequently, the unilateral puncture was also used and the target point was in the anterior one-third middle point of the intervertebral space. The bone entry point was shifted to the cranium and the puncture trajectory was inclined from the posterior cranium to the anterior caudal vertebra. Thus, the inferior endplate could be punctured as intended, and a trajectory from the vertebral body to the disc was prepared. The balloon was then advanced for expansion and then taken out (the cement was not injected in this step).

The modified unilateral transpedicular puncture in steps 1 and 2 was a transverse process-pedicle approach, which was previously described by our surgical team^11^. Briefly, the entry point of the bone surface was localized at the transverse process, 3–5 mm outside the lateral margin of the pedicle projection. The trajectory was from the basilar part of the transverse process to the vertebral body, crossing the pedicle. The puncture and expansion were performed using PKP tools (Kynetyc Medical Technology Co., Ltd., Shanghai, China).

**Figure 3:**
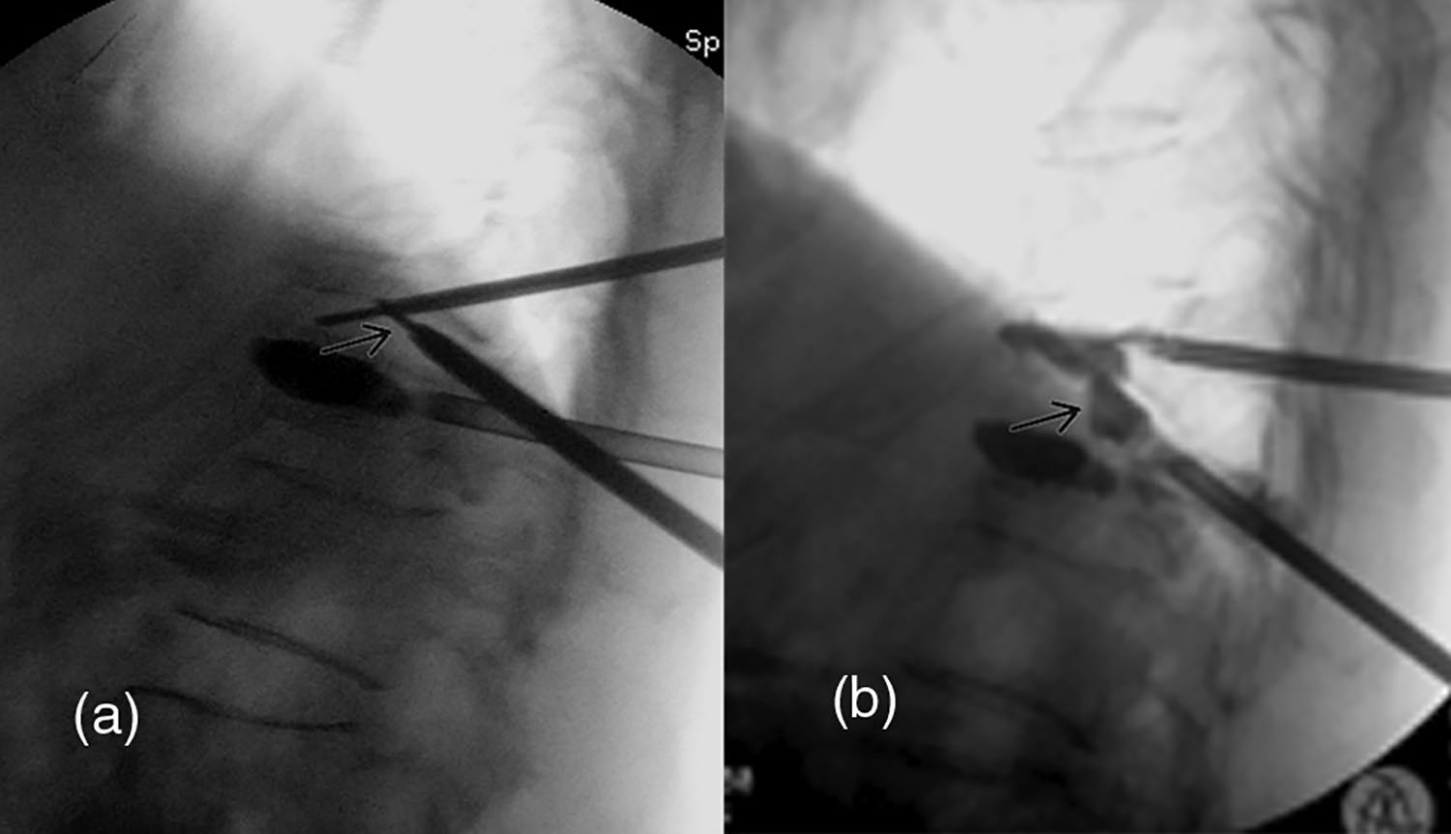
Lateral fluoroscopic images in operation. (**a**) Intervertebral puncture trajectory connected those in the two adjacent vertebrae (arrow); (**b**) cement injection from the cranial and caudal trajectory and the intervertebral trajectory (arrow).

Step 3 was the puncture and expansion of the intervertebral space with EDCI (Fig. 2c). A unilateral extrapedicular puncture was used as follows. The bone entry point was the midpoint of the lateral pedicle of the caudal adjacent fractured vertebra and the target point was the anterior one-third middle point of the cranial adjacent vertebra. The trajectory was from the posterior caudal vertebra to the anterior cranium, crossing the extrapedicle and the intervertebral space. The ideal puncture trajectory in step 3 was a connected channel between the trajectories in steps 1 and 2 (Fig. 3a). Then, the balloon was inflated and the kyphosis was partially corrected.

Step 4 was intervertebral bridging cementoplasty for the unit (Fig. 2d). Lastly, high-viscosity cement (polymethylmethacrylate, PMMA; Heraeus Medical GmbH, Wehrheim, Germany) was prepared and simultaneously injected into the fractured vertebrae and the intervertebral space (Fig. 3b). The two adjacent fractured vertebrae were augmented. Simultaneously, the injured intervertebral space was immobilized with a cement bridge between the two adjacent augmented vertebrae. The injection was carefully monitored with C-arm fluoroscopy to avoid complications of cement leakage. After surgery, routine anti-osteoporosis treatments were used for all patients.

## Results

### Patient demographic and baseline characteristics

Twenty-one female and eleven male patients with an average age of 69.9 years (range 54–93 years) were enrolled. The fractured vertebrae were T11 and T12 in 7 patients, T12 and L1 in 15 patients, and L1 and L2 in 10 patients. The causes in 14 patients were slight life injury from daily activities, such as bending and sneezing, whereas falls and vehicular accident injuries affected 4 and 2 patients, respectively. Twelve patients complained of no trauma. The course was 41.1 ± 45.9 days (range 3–180 days). The bone mineral density (T-Score) was − 3.2 ± − 0.6) (range − 2.5 to − 4.2). The hospital stay duration was 5.6 ± 2.7 days (range 2–15 days) (Table 2).

**Table 2:** Perioperative characteristics of the patients.

Data	Average	Minimum	Maximum
Age (years)	69.9 ± 9.2	54	93
Course (days)	41.1 ± 45.9	3	180
BMD (T-Score)	− 3.2 ± − 0.6	− 2.5	− 4.2
Hospital stay (days)	5.6 ± 2.7	2	15
Operation time (min)	82.8 ± 32.5	40	160
Blood loss (mL)	76.9 ± 31.7	20	150
Cement (mL)	6.4 ± 1.2	4.5	8.0
Follow-up (months)	29.9 ± 13.6	6	54

*BMD* bone mineral density.

### Surgical outcomes

**Figure 4:**
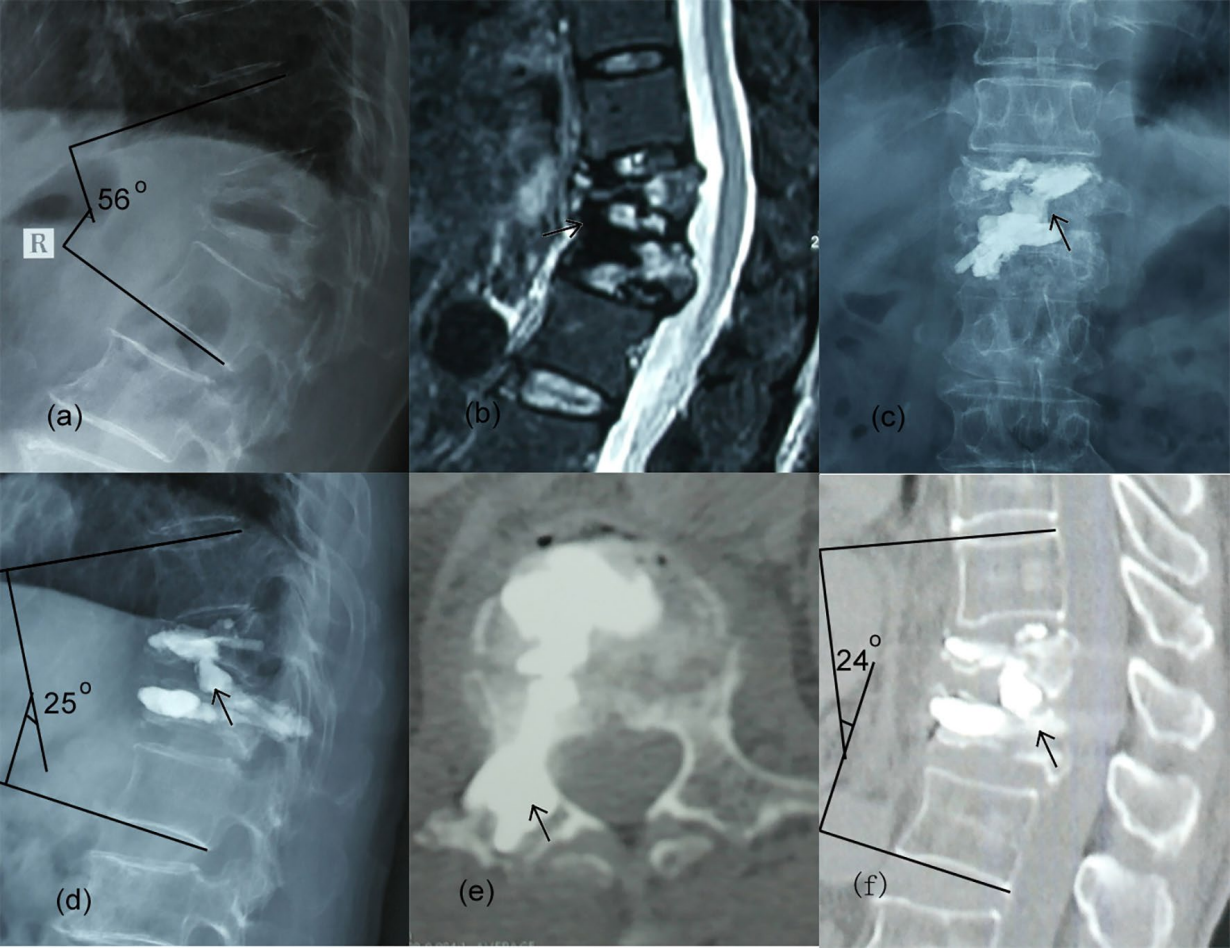
Radiographs of a 73-year-old female with AMOTLFs and EDCI. (**a**) Preoperative lateral radiograph showed T12–L1 severe fractures with a 50° of thoracolumbar kyphotic angle; (**b**) preoperative MRI showed subacute T12 and L1 fractures and the EDCI in T12-L1 (arrow); (**c**) postoperative AP radiograph showed relatively symmetrical cement distribution (arrow); (**d**) postoperative lateral radiograph showed a cement bridge connected between the two adjacent vertebrae and the kyphotic angle decreased to 25° (arrow); (**e**) postoperative CT showed cement leakage in the pedicle (arrow); (**f**) CT reconstruction image in the 2-years follow-up showed the cement bridge was not broke or shifted and the angle sustained well (arrow).

The mean operation time was 82.8 ± 32.5 min. The mean blood loss was 76.9 ± 31.7 mL. The mean injected cement content was 6.4 ± 1.2 mL (Table 2). No intra-operative or postoperative spinal cord or main vessel injury was observed. The cement bridge was a support between the adjacent fractured vertebrae across the intervertebral space; it appeared like a “Z-like” shape when visualized on the lateral X-ray. Cement leakage occurred in 13 patients, with paravertebral leakages in eight patients, disc leakages in four patients, and vessel leakage in one patient. There was no spinal canal cement leakage. A typical case is shown in Fig. 4.

### Follow-up evaluations

The patients were followed up for a mean duration of 29.9 ± 13.6 months. The average VAS score for low back pain was 6.9 ± 0.9 preoperatively, which rapidly decreased to 2.9 ± 0.8 at one day postoperatively and further decreased to 1.7 ± 0.8 at the final follow-up. The average Oswestry disability index was (71.1 ± 7.8)% preoperatively, which decreased to (18.4 ± 5.7)% at one day postoperatively and further decreased to (10.3 ± 5.7)% at the final follow-up. The average thoracolumbar (T10–L2) kyphotic Cobb angle was improved from 46.0° ± 10.4° preoperatively to 25.5° ± 8.5° one day postoperatively, which was seen to be maintained at the final follow-up. Compared with pre-operation, VAS, ODI and Cobb angle were significantly decreased at post-operation 1 day and final follow-up (P < 0.05) (Table 3).

**Table 3:** Parameter assessment at pre-operation, post-operation 1 day and the final follow-up.

Parameter	Pre-operation	Post-operation 1 day	The final follow-up
VAS	6.9 ± 0.9	2.9 ± 0.8*	1.7 ± 0.8*
ODI (%)	71.1 ± 7.8	18.4 ± 5.7*	10.3 ± 5.7*
TLK (°)	46.0 ± 10.4	25.9 ± 8.5*	27.5 ± 7.1*

*VAS* visual analogue scale, *ODI* Oswestry disability index, *TKL* thoracolumbar (T10–L2) kyphotic Cobb angle.

*Compared with the pre-operation using paired *t* test, P < 0.05.

No patients experienced any cement-related adverse event. No incision infection was observed. All patients achieved thoracolumbar stability at the involved level. No cement bridge broke or shifted. Three patients with non-surgical vertebral refracture were readmitted and underwent routine PKP surgery. One patient with postoperative complication of incision hemorrhage was cured by clearance of the hematoma and compression bandage. One patient with urinary infection and three patients with lung infection were treated by antibiotics and recovered.

## Discussion

We herein described a technique of minimally invasive surgery for AMOTLFs with EDCI using PIBC which allowed for augmentation of the fractured vertebrae and immobilization of the adjacent segment simultaneously.

PVP or PKP has been reported as a safe and effective treatment for OVCF^3,4,12^. These traditional cement augmentation techniques have been used for augmentation of the fractured vertebra with the vertebral endplate and disc untreated. As we know, the spinal units include intervertebral disc and the adjacent vertebra. The vertebral endplate-disc complex not only plays a role in allowing motion between adjacent spinal segments but also involved in axial load transfer^13^. An injured vertebral endplate-disc complex may lead to segmental instability of the spinal unit and may predispose the disc to move through a damaged vertebral endplate, which may result in adjacent osteoporotic vertebral fractures^14^. Furthermore, this adjacent vertebral fracture may exacerbate the damage of the involved vertebral endplate-disc complex. The interaction of the adjacent vertebrae and the intervertebral endplate-disc complex may further compromise spinal normal biomechanical properties and may cause or aggravate spinal instability, which may result in chronic vertebrogenic back pain^5^. In addition, cement leakages into the disc are relatively highly frequent in patients with endplate and disc damage. It has been demonstrated that intradiscal cement leak is a significant risk factor for the development of an adjacent vertebral fracture^6,14^. Thus, this stimulated us to try a modified percutaneous puncture technique. Compared to the traditional two levels PVPs or PKPs, PIBC was used for augmentation of the adjacent fractured vertebrae and immobilize the injured intervertebral space simultaneously. This technique was named PIBC by us because of its feature of a cement bridge as a support between the two adjacent augmented vertebrae across the injured intervertebral space.

However, being a combination of puncture, balloon expandation and bridging cementoplasty is technically demanding for PIBC. Among these steps, vertebral puncture and balloon expandation technique have been discussed widely, and some unilateral transpedicular techniques have been described as well^15,16^. Hoh et al.^16^ described a technique using unilateral inflatable balloon tamp via a unilateral transpedicular approach. In our current study, the unilateral transverse process-pedicle technique was used^11^. The bone entry point was shifted to the basilar part of the transverse process, 3–5 mm outside the lateral margin of the pedicle projection, by C-arm fluoroscopy. Thus, the puncture needle could easily and safely meet the midline of the fractured vertebral body and get to the predetermined target point via a unilateral approach. Then, the injected cement could be symmetrically diffused and connected with that from intervertebral puncture. For intervertebral puncture, we tried to use a unilateral extrapedicular approach from the caudal lateral pedicle to the cranial adjacent vertebra. This was because the lumbar pedicle was clear under fluoroscopy, and the operation in the caudal vertebra was more convenient without obstruction from the ribs. For a favoring puncture, acquiring measurements of the related puncture parameters in CT reconstruction images was recommended before operation. Despite this planning, the puncture was a difficult process. But interestingly, it was minimally invasive and feasible. In our study, the average operation time was 82.8 ± 32.5 min, and the average blood loss was 76.9 mL. Chen et al.^17^ reported a mean operation of time 87 min for unilateral kyphoplasty for multilevel OVCFs. Chang et al.^8^ in a technical note of cement augmented-pedicle screw fixation reported that the average operation duration was 4 h 50 min (range 3.5–6 h), and the average blood loss was 421 mL. Our statistics for the operation duration and blood loss volume were comparable to those reported by Chen et al. but were much lesser than those reported by Chang et al.^8,18^. In addition, the PIBC technique was performed under local anesthesia, which makes it suitable for patients with very severe osteoporosis and even those with cardiopulmonary diseases.

In the current study, the clinical results were inspiring. The VAS and the kyphotic angle were decreased obviously and lasted postoperatively, which were similar to those reported in previous literatures^19–21^. In a clinical study, Saxena et al.^20^ demonstrated that the VAS dropped from 6.74 preoperatively to 2.24 postoperatively, and the kyphotic angle was decreased from a preoperative mean angle of 17.41° to a postoperative mean angle of 10.59°. Foo et al.^21^ reported an improvement of 5.0 in VAS and a decrease of 30.77% in kyphotic angle. To improve clinical results, it is important that the trajectory of intervertebral puncture connects with the trajectories of vertebral punctures in the adjacent vertebrae. Thus, the two adjacent vertebrae could be immobilized with a cement bridge. Another important factor was that the puncture should achieve the midline to allow for symmetrical diffusion of the cement^22^. Moreover, complications of injuries of the nerve root, spinal cord, or vessels should be avoided.

Among all the clinical complications, cement leakage was the most common one^4,6^. In our study, cement leakage occurred in 13 patients (40.6%), with most cases of paravertebral and intradiscal leakages. It was possibly closely associated with vertebral endplate-disc injury in adjacent disc space and extrapedicular puncture for intervertebral space. The cement leakage ratio was similar to previous studies about cement augmentation for severe vertebrae fractures^23,24^. In our study, fortunately, the leakages were clinically asymptomatic. Meanwhile, no leakage into the spinal canal was observed, and no cement bridge had broken or shifted until the final follow-up. Three patients with vertebral refracture were largely due to serious osteoporosis. Puncture injuries was an issue for PIBC because of its complex puncture steps. The structures around pedicle (such as nerves, blood vessels, and pleura) may be injured. A definitive predesign of puncture trajectory in preoperation and skillful technique in operation was recommended. To old patients, urinary infection and lung infection were often occurred. Early ambulation was an effective way to decrease these complications. In our study, there was one patient with postoperative incision hemorrhage was due to intercostal vascular injury and cured by clearance of the hematoma. One patient with urinary infection and three patients with lung infection were recovered without adverse consequences. Thus, adverse effects of the technique remained acceptable.

## Conclusions

The current study had limitations of its retrospective nature and small number of cases. In this study, we established that PIBC may be a feasible and effective technique for AMOTLFs with EDCI. This is a minimally invasive surgical technique to augment the fractured vertebrae and immobilize the adjacent segment simultaneously, and we carefully recommend it as an alternative way of pain care for adjacent multilevel osteoporotic thoracolumbar fractures with one intervertebral EDCI.
